# Descemet Membrane Detachment Assessed by Anterior Segment-Optical Coherence Tomography and Managed With Descemetopexy and Corneal Venting Incision: A Case Series

**DOI:** 10.7759/cureus.86631

**Published:** 2025-06-23

**Authors:** Shimpa Kundan, Pramod K Sahu, Akshita Sharma, Gopal K Das, Prince A Aamir

**Affiliations:** 1 Ophthalmology, University College of Medical Sciences & Guru Teg Bahadur Hospital, Delhi, IND

**Keywords:** anterior segment optical coherence tomography (as-oct), complication of cataract surgery, corneal venting incision, descemet membrane detachment, descemetopexy

## Abstract

Descemet membrane detachment (DMD) is a serious complication of cataract surgery noted mostly in the immediate post-operative period. We present a case series of five patients who experienced unresolved corneal edema during the post-operative period with DMD, as observed in slit-lamp examination and confirmed by anterior segment-optical coherence tomography. In all these cases, descemetopexy, along with a corneal venting incision, is performed for better and faster results, resulting in a clearer cornea. Early detection and management of DMD can give excellent visual outcomes and prevent vision loss in such cases.

## Introduction

Descemet membrane detachment (DMD) is a rare iatrogenic complication of cataract surgery with an incidence rate of 2.5% for extracapsular cataract extraction (ECCE) and 0.5% for phacoemulsification [1].

Prior conditions for DMD to occur are blunt instruments, inappropriate AC manipulation, and accidental injection of saline or viscoelastic between the stroma and the Descemet membrane (DM) [2]. There are more chances of DMD in cases performed by trainees [3]. Pre-existing poor endothelial count [4] is also considered a significant risk factor, along with some corneal dystrophies, such as Fuchs corneal endothelial dystrophy and posterior polymorphous corneal dystrophy [3]. An abnormality in the fibrillary stromal attachment to the DM is also a predisposing factor for DMD [5]. Most DMDs are small and involve the peripheral part of the cornea at the incision site and remain clinically insignificant. A total of 0.5% of DMDs are large involving the central part of the cornea, and out of which, 8% require corneal transplantation [6], which is the last resort for DMD according to the HELP algorithm proposed by Kumar et al. [7]. HELP algorithm is a structured approach using anterior segment-optical coherence tomography (AS-OCT) to manage DMD. HELP acronym stands for height, extent, length, and pupil, which are important parameters assessed by AS-OCT to decide the treatment strategy. Descemetopexy is the gold standard for persistent DMD [8,9].

We report a series of five cases who underwent cataract surgery, in which AS-OCT was used to capture images of pre- and post-descemetopexy, along with corneal venting incisions.

## Case presentation

Case 1

**Figure 1:**
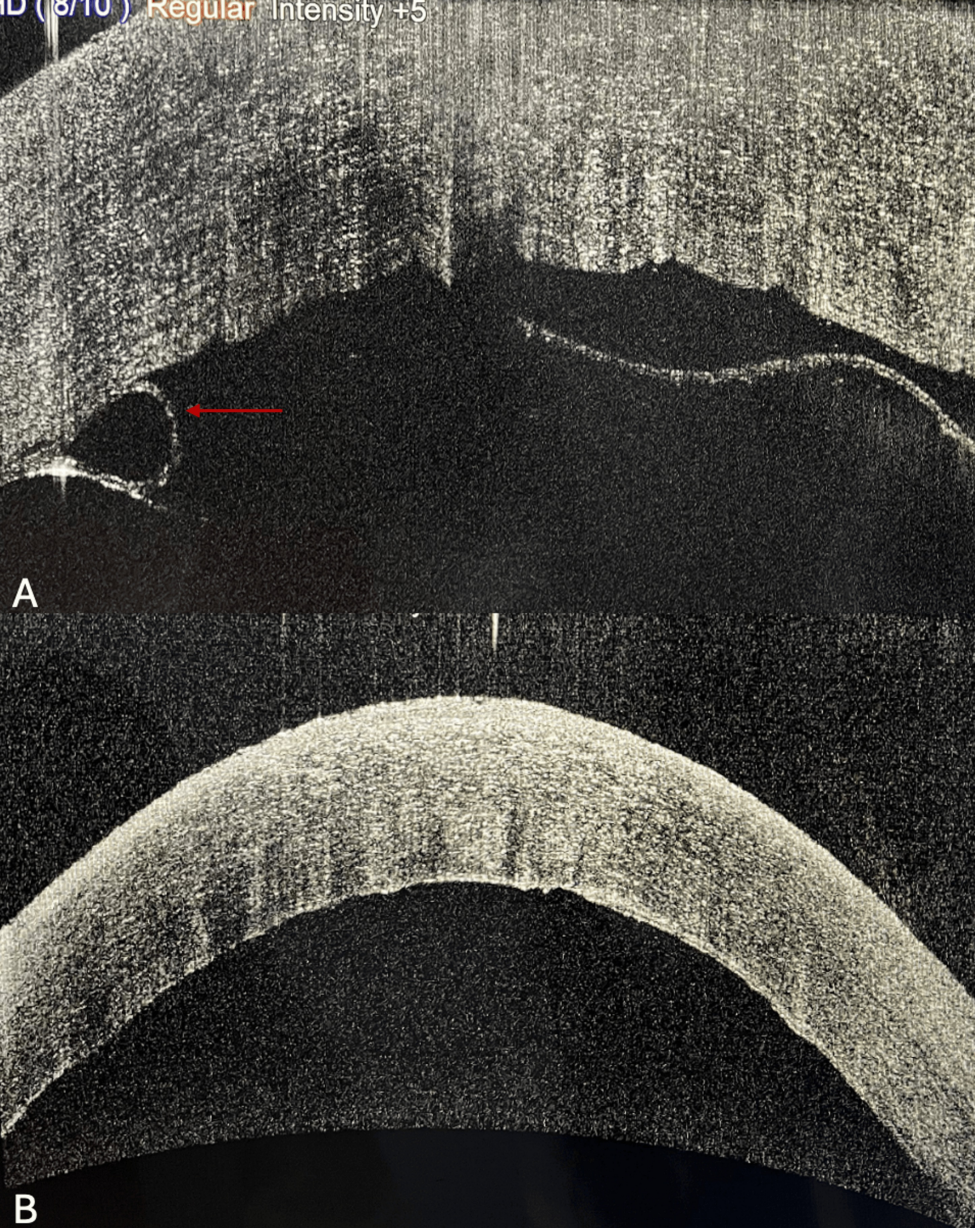
(A) AS-OCT image of Case 1 showing DMD with rolled-up Descemet membrane (red arrow). (B) AS-OCT image after managing DMD with descemetopexy and corneal venting incision. AS-OCT: anterior segment-optical coherence tomography; DMD: Descemet membrane detachment

A 72-year-old male underwent phacoemulsification of his left eye. On the first post-operative day, his best corrected visual acuity (BCVA) was hand movement close to the face (HMCF) with accurate projection of rays in all directions. There was generalized corneal edema. A slit-lamp examination was performed, which revealed a localized DMD at the side port incision (5 o'clock). AS-OCT confirmed our finding, which shows rolled-up DM with fluid pockets. A 14% perfluoropropane (C3F8) gas injection was given under topical anesthesia after making AC paracentesis with the help of a microvitreoretinal (MVR) blade at the limbus superiorly. Immediate post-operative vision was 20/200, which further improved to 20/60 after a week (Figure 1).

Case 2

**Figure 2:**
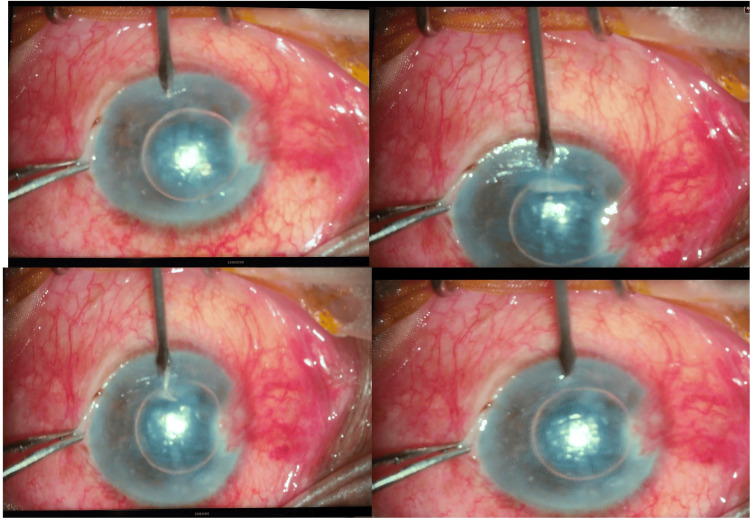
Steps of corneal venting incision in Case 2.

**Figure 3:**
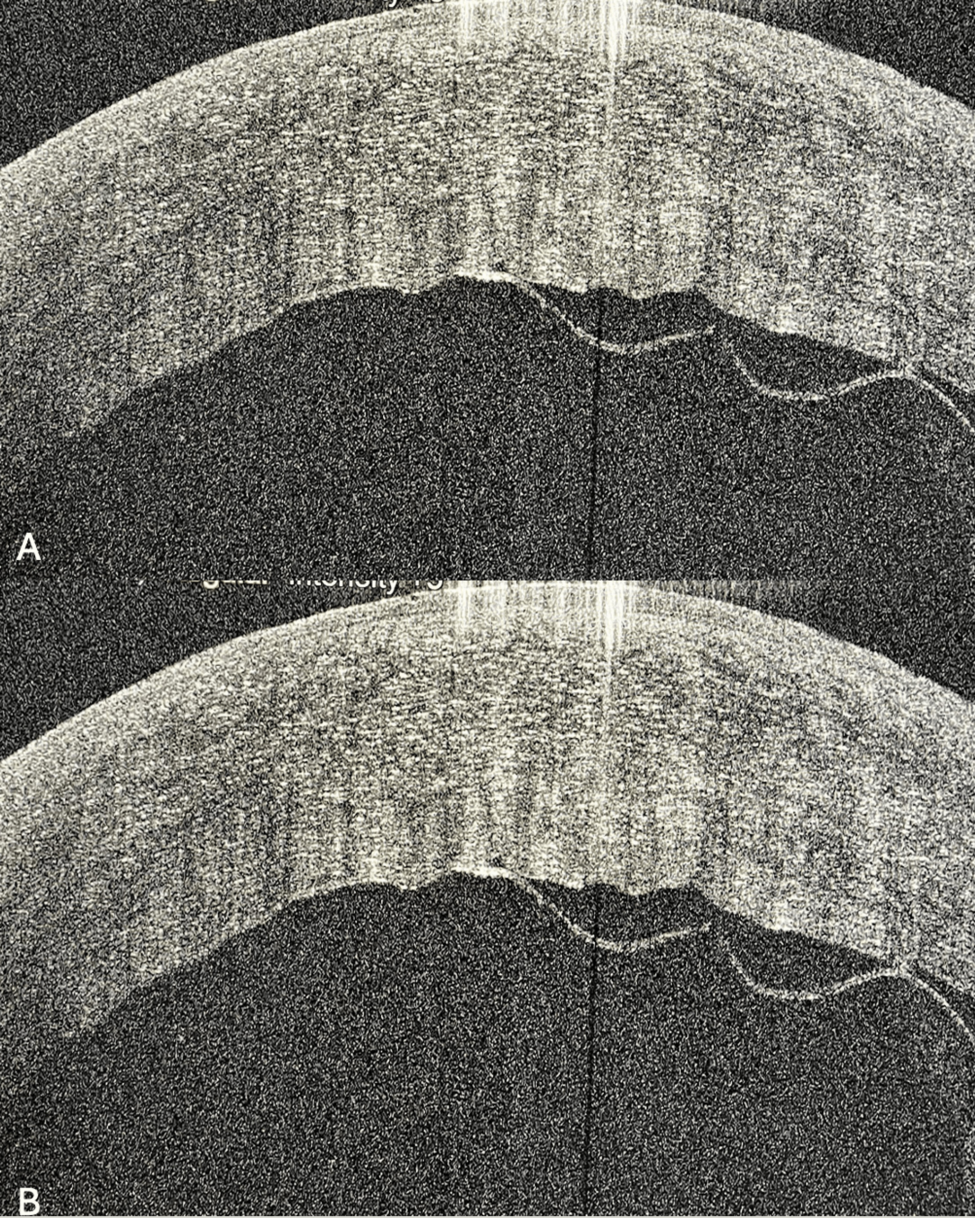
AS-OCT images preoperative (A) and postoperative (B) of Case 2. AS-OCT: anterior segment-optical coherence tomography

An 88-year-old female underwent phacoemulsification right eye. On the first post-operative day, she gained good vision of 20/80. The next day she complained diminution of vision. A slit-lamp examination revealed diffuse microcystic corneal edema. The intraocular pressure (IOP) recorded was 35 mmHg. Slit-lamp examination also revealed localized DMD extending from 6 to 9 o'clock, which was confirmed by AS-OCT. A 20% sulfur hexafluoride (SF6) gas was injected into the AC, followed by a corneal venting incision (Figure 2). Postoperatively, she achieved a visual acuity of 20/80 immediately, which improved to 20/40 after a week (Figure 3).

Case 3

**Figure 4:**
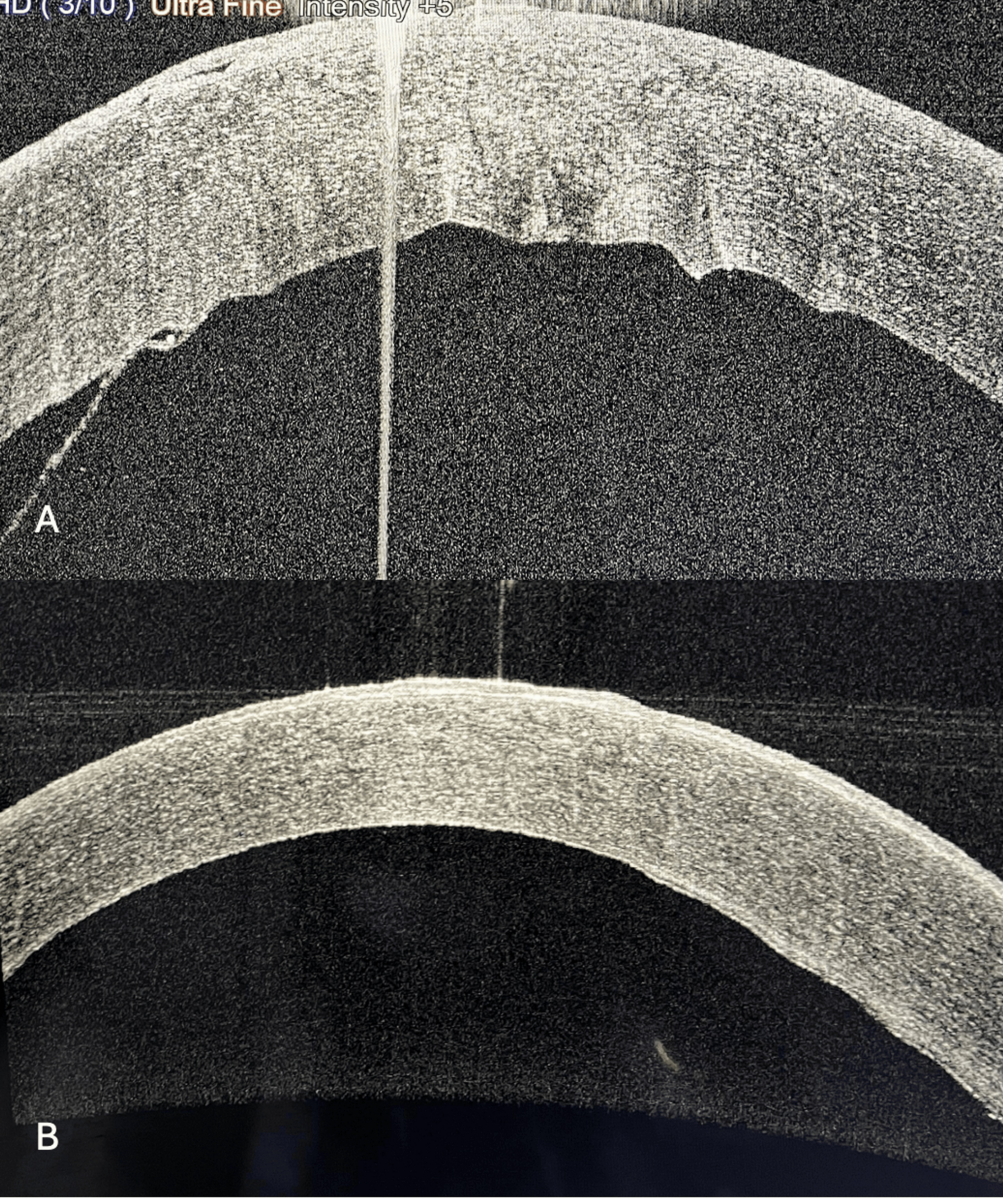
(A) Preoperative AS-OCT image; (B) Postoperative AS-OCT image of Case 3. AS-OCT: anterior segment-optical coherence tomography

A 60-year-old female underwent phacoemulsification in the right eye. On the first post-operative day, she had finger counting close to the face (FCCF) with severe corneal edema along with bullae. Topical steroids and hyperosmotic agents were given. Corneal edema was reduced subsequently, and AS-OCT was performed on day 5 post-operative period. It revealed paracentral DMD. A 14% C3F8 injection was given along with a venting incision. She did not gain significant vision. Her BCVA was 20/200, which further deteriorated to FCCF due to recurrent DMD. She was subsequently managed with corneal transplantation (Figure 4).

Case 4

**Figure 5:**
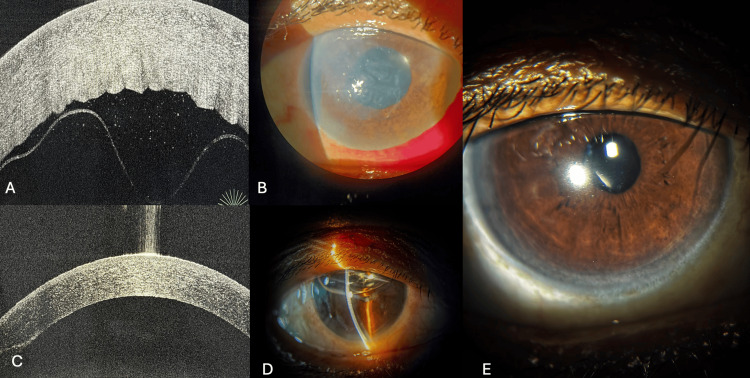
AS-OCT and slit-lamp photographs of Case 4 before and after descemetopexy and corneal venting incision. (A) AS-OCT image showing Descemet membrane detachment (DMD) on day 1 post-cataract surgery. (B) Slit-lamp image showing corneal edema (day 2). (C) AS-OCT image showing attached Descemet membrane post-descemetopexy (day 3). (D) Slit-lamp image showing a clear cornea following descemetopexy with C3F8 and corneal venting incision. (E) Slit-lamp image showing a clear cornea (day 30). AS-OCT: anterior segment-optical coherence tomography; C3F8: perfluoropropane

A 62-year-old female underwent right eye small-incision cataract surgery (SICS). On post-operative day 1, she gained the vision of 20/30. The same evening, she complained gradual diminution of vision, and her vision deteriorated to 20/200. A slit-lamp examination revealed corneal edema along with inferior DMD. AS-OCT was done to confirm and to look for maximum fluid pockets. A 14% C3F8 was injected, and a corneal venting incision was performed. Her vision improved the next day to 20/80. Her BCVA during post-procedure was 20/40, which further improved to 20/30 (Figure 5).

Case 5

**Figure 6:**
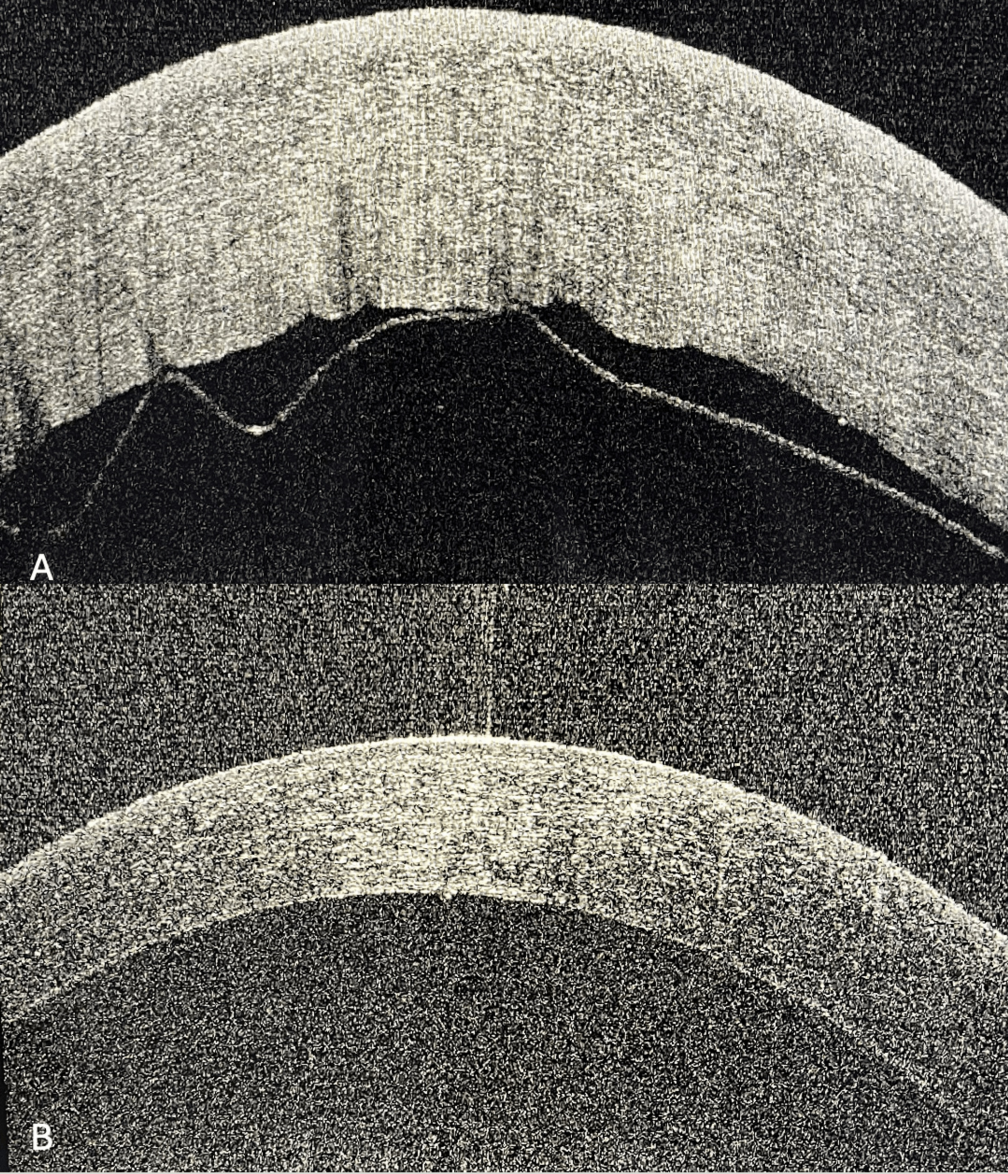
(A, B) AS-OCT images of Case 5 showing pre- and post-descemetopexy status. AS-OCT: anterior segment-optical coherence tomography

A 55-year-old female underwent left eye ECCE. Intraoperative DMD was noticed in the inferior quadrant. Air tamponading was done, and the case closed. The post-operative cornea was clear on day 1. Post-operative day 2 showed diffuse corneal edema along with DMD under slit-lamp examination. Her vision reduced from 20/30 to HMCF. AS-OCT was done for confirmation. A similar procedure was done, and corneal edema was reduced, and she gained good vision (Figure 6).

In all these cases, the procedure followed was similar. The AC was filled with 14% C3F8 or 20% SF6, after which a limbal paracentesis was made by MVR blade located mostly opposite to the detachment site. After this, a 23-gauge needle is used to puncture the peripheral cornea at the area of greatest fluid depth to drain pre-descematic fluid. This fluid oozes out from the partial thickness made in the cornea. This thickness was made up of the stromal layer of the cornea. Table 1 shows a summary of all five cases, including the type of surgery, the type of gas used, and pre- and post-procedure BCVA.

**Table 1:** Summary of all five cases including type of surgery, time of DMD detection, timing of AS-OCT, type of gas used, pre- and post-procedure BCVA. BCVA: best corrected visual acuity; POD: post-operative day; Phaco: phacoemulsification; HMCF: hand movement close to the face; C3F8: perfluoropropane; SF6: sulphur hexafluoride; FCCF: finger counting close to the face; SICS: small incision cataract surgery; ECCE: extracapsular cataract extraction; DMD: Descemet membrane detachment; AS-OCT: anterior segment-optical coherence tomography

Case	Age/sex of the patient	Eye	Type of cataract surgery	BCVA before descemetopexy and corneal venting incision	Time of DMD detection	Timing of AS-OCT	Gas used	BCVA after descemetopexy and corneal venting incision	Final BCVA after a week
1.	72/Male	Left	Phaco	HMCF	POD1	POD 2	14% C3F8	20/200	20/60
2.	88/Female	Right	Phaco	20/80	POD2	POD 2	20% SF6	20/80	20/40
3.	60/Female	Right	Phaco	FCCF	POD1	POD 5	14% C3F8	20/200	FCCF
4.	62/Female	Right	SICS	20/200	POD1	POD 3	14% C3F8	20/40	20/30
5.	55/Female	Left	ECCE	HMCF	POD2	POD 3	14% C3F8	FCCF	20/200

Post this procedure, topical corticosteroids and antibiotics were continued, and the patients were asked to lie down supine for 24 hours. The next day, AS-OCT was done to note down the attachment of DM to the stroma. All these cases were followed for a month, except Case 3 for two weeks, which had recurrent DMD and was suggested to undergo corneal transplantation.

## Discussion

This case series highlights the management of DMD after cataract surgery with the help of a tamponading agent in isoexpansile concentrations such as 14% C3F8 or 20% SF6 along with corneal venting incisions. A similar procedure was earlier done by Ghaffariyeh et al. [10], Weng et al. [11], and Bhatia and Gupta [12]. The main principle of corneal venting incision is to drain the entrapped fluid in the supra-Descemet’s space that otherwise prevents the apposition of DM to stroma even after descemetopexy. Corneal venting incisions were primarily used in Descemet’s stripping endothelial keratoplasty (DSEK) [13]. Incidence of DMD following phacoemulsification is around 0.04%, which is lower than that compared to other cataract surgeries [3].

Schiempflug imaging and AS-OCT are important diagnostic tools, other than slit-lamp examination, to delineate the extent of DMD [14,15]. It also helps in differentiating cases with dense corneal edema to pick up any DMD and cases of pseudo-phakic or aphakic bullous keratopathy. DMD was earlier classified by Mackool and Holtz [6], and a recent classification based on AS-OCT by Kumar et al. [7].

Center involving DMD needs urgent management, and if left untreated, can lead to corneal scarring [16] as well as bullous keratopathy, which can cause pain and increase the risk of microbial keratitis. Most of the DMDs involve the peripheral cornea and do not require any surgical intervention. These DMDs can be managed with topical corticosteroids and hyperosmotic agents, with a spontaneous reattachment rate of around 60% [17].

Disadvantages of descemetopexy are persistent DMD, corneal decompensation, appositional angle closure (18%), pupillary block (2.1%), and uveitis (2.7%) in a retrospective study of 112 patients done by Odayappan et al. [3]. The disadvantages are mostly associated with the injection of a large amount of gas. These disadvantages can be overcome by combining descemetopexy with corneal venting incisions [3]. The drawbacks of corneal venting incisions include corneal scarring and induced astigmatism. These can be minimized by carefully planning the incision sites at the highest point of DMD while avoiding the visual axis [11]. If all interventions fail, keratoplasty may be required to restore vision.

Our study is different from other studies because we have a case report of five cases. Bhatia and Gupta [12], Ghaffariyeh et al. [10], Weng et al. [11], and Menezo et al. [18] have studied a single case, and after failed attempts of pneumatic descemetopexy, corneal venting along with descemetopexy was tried. In our case series, we have considered treating DMD with descemetopexy along with corneal venting incisions in the first attempt. This gave us better results immediately after surgery with lower failure rates. Out of five cases, only one case did not gain vision due to a delay in management. One case reported reduced vision due to astigmatism caused by corneal sutures following ECCE surgery. Most of the patients gained good vision within a week in comparison to previous literature describing a mean duration of 16.0 ± 7.1 days to resolution of DMD [12]. The gain in good vision within a week in our study was due to early intervention in the management of DMD, and the height of DMD being smaller in our cases.

Our technique is superior because of minimal instrumentation and earlier attachment of DM, but also has a risk of high IOP due to the use of long-acting gases such as SF6 and C3F8.

## Conclusions

DMD is one of the rare complications of cataract surgery. Better outcomes of DMD have been seen if treated immediately and effectively. AS-OCT can be used for diagnosing DMD in cases of severe corneal edema. Descemetopexy, along with corneal venting incisions, is an effective method for the management of DMD. The use of modern technology, such as AS-OCT, helps in deciding the further management of DMD with the HELP algorithm. Descemetopexy, when combined with corneal venting incision, turns out to be a quicker and minimally invasive technique for the treatment of DMD. A larger sample size is required to make our study more conclusive for the management of DMD.
